# Influence of Psychological Safety and Safety Climate Perceptions on Nurses’ Infection Prevention and Occupational Safety Practices and Environment

**DOI:** 10.3390/nursrep15020037

**Published:** 2025-01-24

**Authors:** Cara Thurman Johnson, Nancy Spear Owen, Amanda J. Hessels

**Affiliations:** 1 Columbia University School of Nursing, 560 W 168th St., New York, NY 10032, USA; cbt2119@caa.columbia.edu (C.T.J.); nowen@pace.edu (N.S.O.); 2 North Carolina Translational and Clinical Sciences Institute, University of North Carolina at Chapel Hill School of Medicine, 160 N, Medical Drive, Chapel Hill, NC 27599, USA; 3 Center for Excellence in Healthcare Simulation, College of Health Professions, Pace University, 861 Bedford Road, Pleasantville, New York, NY 10570, USA; 4 Hackensack Meridian Health Ann May Center for Nursing and Allied Health, 2020 6th Avenue, Neptune City, NJ 07753, USA

**Keywords:** psychological safety, infection prevention, patient safety, occupational health, nursing, burnout, safety culture

## Abstract

**Background/Objectives**: The aim of this study was to describe and examine the relationships among elements of infection prevention practices, the care environment, psychological safety, and safety climate in adult medical surgical units in the wake of the COVID-19 pandemic. **Methods**: Nurses in adult inpatient medical surgical units in the northeast were surveyed electronically. Each self-rated their infection prevention practices and elements of the care environment in their primary work unit. They were also asked to rate a series of questions regarding how psychologically safe they felt on their units as well as the overall patient safety climate. **Results**: A total of 259 nurses responded (52% response rate) to the survey. Overall psychological safety was rated neutrally among respondents, with a rating of 3.5 (1 being the lowest and 5 being the highest). Respondents reported better ratings of the safety climate on their unit (4.0) but also identified areas for improvement. Eight of twelve infection prevention practices were correlated with higher safety climate scores and ten were correlated with higher psychological safety scores. Nine of ten environmental factors were correlated with higher safety climate and higher psychological safety scores. **Conclusions**: Both psychological safety and patient safety climate are related to nurse self-ratings of performance of infection prevention practices. Similarly, the care environment nurses work in has important implications for psychological safety and patient safety. It is essential for nursing leadership to act as a steward in these areas to build a higher quality care environment for nurses and patients alike.

## 1. Introduction

Fostering a culture of patient safety in US hospitals is an important predictor of many healthcare quality metrics such as healthcare-associated infections and adherence to standard precautions among direct care clinical staff [1]. A key aspect of patient safety culture is the perceived psychological safety and safety climate of clinical team members as a collective group [2,3]. Psychological safety is defined as the degree to which employees perceive they will not be penalized for speaking up, raising questions, asking for help, sharing ideas, making mistakes, or expressing concerns [3,4,5]. In clinical care team dynamics where there are roles that are perceived as lower or higher status, psychological safety can mitigate barriers to communication between status levels [6]. Higher levels of psychological safety at the group level have been shown in the literature to improve error reporting and infection prevention practices to reduce healthcare-associated infections, such as nurse-initiated catheter discontinuations and sedation vacations [5]. Psychological safety is an aspect of the clinical care environment that must be continuously fostered, especially in times of crisis [2,3,7,8]. These key interpersonal constructs are often studied in isolation despite the unique importance of both safety culture and psychological safety to optimize organizational behavior, worker performance, and outcomes [2].

During the past three years, the COVID-19 pandemic has repeatedly and protractedly put the acute healthcare system in a state of crisis, placing numerous physical and psychological stressors on clinical nurses [9,10]. Overcrowded hospitals, shortages of essential PPE, long working hours, and staffing shortages were all shockwaves in the care environment that nurses were required to navigate, often without proper support and communication from leadership [10,11,12].

The northeast US was the epicenter of COVID-19 during the spring of 2020 when the pandemic first began to spread in the United States [13]. During this time, hospitals were struggling to keep up with the number of patients needing critical care for COVID-19 [14,15,16]. Meeting surge capacity also meant a substantial increase in staffing needs. Nurses were redeployed locally, recalled from retirement, and recruited from other locations in the US, temporary staffing agencies, and the Department of Defense [17]. Moreover, frontline healthcare providers were at an increased risk of catching COVID-19, three times more likely than the general population [12]. In the first year of the pandemic, over 3600 healthcare workers were removed from the workforce through COVID-19 deaths alone [12]. New York City hospitals remained majorly impacted across the multiple waves of COVID-19 variants, causing repeated stressors to bedside care nurses and potentially impacting the hospital and unit culture [18,19,20]. The negative impact that COVID-19 has had on the emotional well-being, mental health, and feelings of burnout in bedside nurses is well documented in the literature [21,22]. This cross-sectional, descriptive study is part of a larger ongoing research trial, the Simulation to Improve Infection Prevention and Patient Safety (SIPPS) Trial (AHRQ R18HS26418). The parent study is a 5-year group-randomized, group-interventional simulation trial taking place in two hospitals in two northeastern states. The aims of this study are to (1) describe the perceptions of safety climate and psychological safety; and (2) examine relationships among self-reported infection prevention and occupational safety behaviors and the environment of acute care nurses practicing in hospitals two years post-COVID-19 emergence in the US.

## 2. Materials and Methods

Participants were recruited across 2 major metropolitan medical centers in the tristate area from 6 units per site (12 total) to complete a baseline survey towards the beginning of the study period. The inclusion criteria were nurses who provided at least 16 h per week of direct patient care and had worked at the hospital for six months or more. This study was approved by IRB at both participating sites prior to the recruitment of research subjects. Anonymous electronic survey invitations were distributed through work listservs, and all data were collected between February 2022 and April 2022. A USD 25 Amazon electronic gift card was provided as an incentive for participation.

The 38-item survey assessed four domains: (1) patient safety climate (9 items); (2) psychological safety (7 items); (3) self-reported infection prevention and occupational safety behaviors (12 items); and (4) the unit infection prevention and occupational safety environment (10 items). Safety climate was measured using items from the Safety Organizing Scale, assessing individual nurse perceptions of team behavior and safety climate in their hospital units [23]. The nine items are scored on a scale from 1 = “Not at all” to 5 = “To a great extent”. Items are mapped to the five principles of high-reliability organizational and mindfulness constructs: (1) preoccupation with failure; (2) reluctance to simplify interpretations; (3) sensitivity to operations; (4) commitment to resilience; and (5) deference to expertise [23].

For the second domain, psychological safety, nurses rated how frequently they experience seven conditions indicative of psychological safety in their work environment, their perceptions that they can engage in risk-taking with their team, from 1 = “Never” to 5 = “Always” [4]. Two items were reverse-coded in the analysis as the prompts were negatively phrased.

The third domain, infection prevention and occupational safety practices, asked nurses how often they performed 12 infection prevention and occupational safety behaviors from 1 = “Never” to 5 = “Always” [24]. Similarly, for the fourth domain, nurses also self-reported how conducive the work environment was to these practices. They were asked how much they agreed or disagreed with 10 statements about their primary unit from 1 = “Strongly Disagree” to 5 = “Strongly Agree” [24]. Negatively phrased statements were reverse-coded for analysis.

At the end of the survey, nurses were asked several demographic items including items such as sex, age group, shift worked, tenure on unit, certification in specialty, and patient load.

Descriptive statistics and Pearson’s correlation coefficient testing were performed using SAS^®^ 9.4 with a significance level set at *p* < 0.05 [25]. The survey consisted of 38 items, and Cronbach’s alpha demonstrated excellent internal reliability (*α* = 0.90).

## 3. Results

### 3.1. Response Rate and Demographics

The response rate was 52% with 311 total responses, and the final analytical sample included 259, excluding incomplete surveys. Demographic data are shown in Table 1.

#### 3.1.1. Domain Descriptives

##### Safety Climate

The mean safety rating was 4.0. The item nurses rated the highest was “*When giving report to an oncoming nurse, we usually discuss what to look out for*” (*M* = 4.4); the lowest-rated item was “*We discuss alternatives as to how to go about our normal work activities*” (*M* = 3.6). The nine items measuring safety climate were further divided into five collective mindfulness subdimensions. In descending order, nurses rated deference to expertise (*M* = 4.2), preoccupation with failure (*M* = 4.1), commitment to resilience (*M* = 3.9), sensitivity to operations (*M* = 3.8), and reluctance to simplify interpretations (*M* = 3.6).

##### Psychological Safety

The mean psychological safety rating was 3.5. The item rated the highest was “*When a medical error occurs at this hospital, health care workers are encouraged to discuss mistakes in order to learn how to prevent similar future errors*” (*M* = 4.0); the lowest-rated item was “*At this hospital, people are too busy to invest time in improvement*” (*M* = 3.1).

##### Infection Prevention and Occupational Safety Practices

Nurses rated their performance of most infection prevention and occupational safety practices very highly, with a mean score of 4.5. The highest-rated item was “*Immediately dispose of sharp objects into a sharps container*” (*M* = 4.9); the lowest-rated statement was how often they “*Omit wearing a face shield or goggles when there is a possibility of a splash, spray, or splatter of patient bodily fluids to my eyes*” (*M* = 3.8).

##### Infection Prevention and Occupational Safety Environment

Nurses rated their work environment as modestly conducive to infection prevention and occupational safety with a mean score of 3.9. The highest-rated statement was “*If a patient on my unit is identified as needing isolation precautions (droplet, airborne, or contact) appropriate actions are taken immediately*” (*M* = 4.4). Although this item had the highest rating, nurses still reported not having adequate space to isolate patients when the need arises, with only 56% reporting often or always having it available. The lowest-rated statement was “*My work area is not crowded with people*” (*M* = 3.3).

#### 3.1.2. Correlations Among Organizational Safety Perceptions, Infection Prevention, and Occupational Safety Practices and Environment

The mean safety climate and psychological safety domain scores were statistically significantly correlated with most infection prevention and occupational safety self-reported practices and environment (Table 2). Eight of twelve practices were correlated with higher safety climate scores and ten of twelve practices were correlated with higher psychological safety scores. Two practices were neither correlated with safety climate nor psychological safety, “Wear a disposable face mask whenever there is a possibility of a splash, spray, or splatter of patient bodily fluids to my nose or mouth” and “Eat or drink in an area where there is a possibility of exposure to or becoming with blood or body fluids”. Nine of ten environmental factors were correlated with higher safety climate and higher psychological safety scores. One factor was not associated with either safety score: “My primary job duties often interfere with my being able to follow standard precautions”.

## 4. Discussion

Ensuring that patient safety and infection prevention practices are reliably practiced is routinely challenging at the bedside and became notably more complex with the pandemic as additional precautions were necessitated and care environments became increasingly laden with technology, patient density swelled, and team functioning strained [26]. These overwhelming events prompted the need to revisit the relationships among medical surgical nurses’ perceptions of psychological safety, patient safety climate, infection prevention and occupational practices and environments. This study identified that nurses’ perception of psychological safety was neutral and the lowest rated of all survey domains. Perceptions of the infection prevention and occupational safety environment and the patient safety climate were slightly higher, and the most positive perception was infection prevention and occupational safety practices.

The findings indicate that even though medical surgical nurses only sometimes felt psychologically safe, they did report high infection prevention and safety practices. It is important to highlight that despite statistical significance, the relationship between psychological safety and infection prevention behaviors was somewhat weak, explaining 11–30% of the variance in behaviors. This differs with findings reported prior to the pandemic where a national survey study by Greene, Gilmartin, and Saint, 2020, found that psychological safety was rated high and associated with three infection prevention behaviors related to urinary tract infection prevention and ventilator-associated pneumonia prevention [5]. This may in part be due to the behaviors measured in our survey including general infection prevention and occupational safety practices rather than device-specific prevention practices.

These findings also add to the larger body of literature on psychological safety by identifying the specific characteristics of leadership that nurses rate as pivotal to higher levels of reported adherence to infection prevention and occupational safety. Ma et al., 2021, conducted a study during the pandemic, finding that a strong servant leadership style mediated by psychological safety helped to prevent nurse burnout [27]. Our results further this knowledge and indicate that post-pandemic, there is work to be done by nursing leadership in hospitals to ensure a safe work environment where nurses speak up, report mistakes, address difficult problems on the unit, and feel comfortable correcting errors [27,28]. Results also highlight that refreshers of psychological safety skill-building exercises and educational activities may be needed following an infectious disease crisis. This study supports the current literature on post-pandemic healthcare.

A major finding of this study is the perceptions of safety climate as mapped to high-reliability mindfulness subdimensions. Despite lower perceptions of psychological safety, nurses rated deference to expertise highest, followed by preoccupation with failure, commitment to resilience, sensitivity to operations, and finally reluctance to simplify interpretations. The poor ratings to prevent oversimplification indicate a lack of willingness of leaders to challenge perceptions of the way “things are done” and acknowledge the complexity of a situation [28]. This provides insight into the importance of the organization being responsive and agile in dynamic and evolving workplace conditions, such as providing direct nursing care in hospitals during a pandemic. This is consistent with the theoretical contributions of Saleem et al. (2021), who posit workforce agility as an antecedent for mindful organizing, and in turn safety performance [29].

As both safety climate and psychological safety were found to be directly correlated with infection prevention and occupational safety practices and behaviors, it would follow that organizational commitment and action to enhance the psychological safety of providers and patient safety climate have the potential to improve healthcare worker and patient outcomes. Further, our study identified a moderate positive relationship between infection prevention and occupational safety environment and the units’ safety climate as well as psychological safety. This may add value to the development of future training or hospital initiatives to minimize hazards and protect workers. The survey item assessing support from management in preventing transmissible occupational health hazards had the highest correlation with both psychological safety and safety climate, explaining over 50% of the variance in responses, shining a key light on ways in which nursing leadership can build a safer and better care environment for nurses and therefore patients.

### 4.1. Limitations

As with all studies, ours had limitations that should be considered. These include the use of a self-report instrument which has potential for participant bias and social desirability for positive self-report of behaviors. Strategies to mitigate this bias include making the survey voluntary and anonymous, and using a reliable survey instrument. We also acknowledge that the study was conducted at two different hospitals in the northeast United States, and this may limit generalizability. This was a descriptive study, in which we did not aim to analyze differences between groups of our population. Comparisons were not made between the varying lengths of service of healthcare staff, as well as other demographic characteristics, which could lend greater insight into the findings.

### 4.2. Conclusions

Psychological safety and patient safety climate are related to nurses’ self-assessments of their infection prevention and occupational safety practices. Implications for future research include the need to build and enhance psychological safety as part of infection prevention and patient safety initiatives and explore in greater depth the perceived benefit of such actions from the perspective of frontline nurses. Potential implementation science trials and evaluations might evaluate methods to support hospital nurses before, during, and after a crisis, examine leadership styles, and develop continuous training programs. Policy and practice implications in the short and near term are evident. It is vital that researchers and leaders not only survey but actively listen to frontline staff, and in turn that frontline staff speak up and out about concerns, authentically and with a forward-looking lens. It is crucial for nursing leadership to act as stewards in these areas, fostering a higher-quality care environment for both nurses and patients. This can happen if policymakers, private and public healthcare funders, and organizational leaders place value and create soft and hard metrics and initiatives to ensure these crucial conversations are had, and create the environment, including training, to ensure the skills required to perform safely in the chaos of modern healthcare are optimized.

## Figures and Tables

**Table 1:** Demographic characteristics.

Characteristic	*n*	*%*
**Time at Hospital**		
<1 year	38	12
1 to 5 years	109	35
6 to 10 years	27	9
11 to 15 years	13	4
16 to 20 years	20	6
>20 years	12	4
**Position**		
Staff Registered Nurse	195	63
Other	7	2
Nurse manager, assistant manager	14	5
**Work Status**		
Full-time	206	66
Part-time	7	2
Per diem or travel	5	2
**Highest Degree**		
RN diploma	16	5
Master’s degree	31	10
ADN	14	5
BSN	148	48
Bachelor’s degree outside of nursing	6	2
**National Certification**		
Yes	88	28
No	130	42
**Time in Specialty**		
<1 year	37	12
1 to 5 years	102	33
6 to 10 years	31	10
11 to 15 years	17	5
16 to 20 years	16	5
>20 years	15	5
**Gender**		
Female	195	63
Male	22	7
**Age (years)**		
<25	22	7
25–34	108	35
35–44	40	13
45–54	30	10
55–64	14	5
**Shift**		
Days (8 or 12 h)	106	34
Evenings (8 or 12 h)	5	2
Nights (8 or 12 h)	105	34

Notes: Responses may not total 259 due to missing data. Response categories with fewer than five respondents are not included.

**Table 2:** Associations among safety climate, psychological safety, infection prevention, and occupational safety practices and environment (n = 259).

Practice and Environment Items	Rating	Safety Climate		Psychological Safety	
**Infection Prevention and Occupational Safety Practices**	**Mean (SD)**	**r**	*p* *	**r**	*p* *
“Immediately dispose of sharp objects into a sharps container”.	4.9 (0.3)	0.15	0.02 *	0.16	0.01 *
“Dispose of all potentially contaminated materials into a red (and/or labeled) bag for disposal as biomedical waste”.	4.8 (0.4)	0.13	0.04 *	0.15	0.02 *
“Wear disposable gloves whenever there is a possibility of exposure to blood or other bodily fluids”.	4.9 (0.3)	0.20	0.002 *	0.18	0.004 *
“Eat or drink in an area where there is a possibility of exposure to or becoming with blood or body fluids”.	4.0 (1.5)	−0.08	0.9	0.11	0.09
“Recap needles because it is unavoidable where I work”.	3.9 (1.4)	0.06	0.35	0.14	0.03 *
“Immediately place soiled or contaminated linens in soiled linen bin”.	4.6 (0.6)	0.30	<0.001 *	0.29	<0.001 *
“Assess patient care activities risk for the possibility of a splash, splatter, or spray from a patients’ bodily fluids to my nose, mouth, or eyes”.	4.6 (0.6)	0.24	<0.001 *	0.23	<0.001 *
“Wipe all potentially contaminated surfaces with approved disinfectant after patient care”.	4.6 (0.7)	0.27	<0.001 *	0.30	<0.001 *
“Perform hand hygiene before touching a patient”.	4.8 (0.4)	0.22	<0.001 *	0.25	<0.001 *
“Wear an outer garment that is resistant to blood and bodily fluids when there is a chance of soiling/contaminating my clothes”.	4.5 (0.8)	0.18	0.005 *	0.21	<0.001 *
“Omit wearing a face shield or goggles when there is a possibility of a splash, spray, or splatter of patient bodily fluids to my eyes”.	3.8 (1.5)	0.07	0.24	0.19	0.003 *
“Wear a disposable face mask whenever there is a possibility of a splash, spray, or splatter of patient bodily fluids to my nose or mouth”.	4.8 (0.6)	0.01	0.89	-0.03	0.65
**Infection Prevention and Occupational Safety Environment**	**Mean (SD)**	**r**	*p* *	**r**	*p* *
“My primary job duties often interfere with my being able to follow standard precautions”.	3.7 (1.2)	0.09	0.15	0.29	0.30
“My work area is not crowded with people”.	3.3 (1.1)	0.26	<0.001 *	0.32	<0.001 *
“I usually have too much to do and can’t always follow standard precautions”.	3.6 (1.1)	0.23	<0.001 *	0.40	<0.001 *
“The protection of workers from occupational exposures to HIV is a high priority with management where I work”.	4.1 (0.9)	0.42	<0.001 *	0.50	<0.001 *
“On my unit, all reasonable steps are taken to minimize hazardous job tasks and procedures”.	4.1 (0.8)	0.54	<0.001 *	0.56	<0.001 *
“Employees are encouraged to become involved in safety improvements”.	4.3 (0.8)	0.49	<0.001 *	0.58	<0.001 *
“Managers on my unit do their part to insure employees are protected from occupational blood-borne pathogens and communicable diseases”.	4.2 (0.8)	0.56	<0.001 *	0.59	<0.001 *
“My work area is kept clean”.	4.0 (1.0)	0.41	<0.001 *	0.42	<0.001 *
“My work area is cluttered with items and objects”.	3.3 (1.2)	0.17	0.01 *	0.28	<0.001 *
“On my unit, we have enough personal protective equipment if needed to follow standard precautions”.	4.3 (0.8)	0.38	<0.001 *	0.41	<0.001 *

Notes: Safety climate and psychological safety measured as mean score of responses; bold * = statistically significant at *p* ≤ 0.05.

## Data Availability

The datasets presented in this article are not readily available because the data are part of an ongoing study.

## Public Involvement Statement

No public involvement in any aspect of this research.

## Guidelines and Standards Statement

This manuscript was drafted using the STROBE guidelines for cross-sectional studies by von Elm E, Altman DG, Egger M, Pocock SJ, Gotzsche PC, and Vandenbroucke JP. Strengthening the Reporting of Observational Studies in Epidemiology (STROBE) Statement: guidelines for reporting observational studies (2007).

## References

[1] Hessels A.J., Guo J., Johnson C.T., Larson E. (2023). Impact of patient safety climate on infection prevention practices and healthcare worker and patient outcomes. Am. J. Infect. Control.

[2] Edmondson A.C., Lei Z. (2014). Psychological Safety: The History, Renaissance, and Future of an Interpersonal Construct. Annu. Rev. Organ. Psychol. Organ. Behav..

[3] Gilmartin H.M., Langner P., Gokhale M., Osatuke K., Hasselbeck R., Maddox T.M., Battaglia C. (2018). Relationship Between Psychological Safety and Reporting Nonadherence to a Safety Checklist. J. Nurs. Care Qual..

[4] Edmondson A. (1999). Psychological Safety and Learning Behavior in Work Teams. Adm. Sci. Q..

[5] Greene M.T., Gilmartin H.M., Saint S. (2020). Psychological safety and infection prevention practices: Results from a national survey. Am. J. Infect. Control.

[6] Nembhard I.M., Edmondson A.C. (2006). Making it safe: The effects of leader inclusiveness and professional status on psychological safety and improvement efforts in health care teams. J. Organ. Behav..

[7] Devaraj L.R., Cooper C., Begin A.S. (2021). Creating Psychological Safety on Medical Teams in Times of Crisis. J. Hosp. Med..

[8] Porter T.H., Peck J.A., Bolwell B., Stoller J.K. (2023). Authentic leadership at the Cleveland Clinic: Psychological safety in the midst of crisis. BMJ Lead..

[9] Arnetz J.E., Goetz C.M., Arnetz B.B., Arble E. (2020). Nurse Reports of Stressful Situations during the COVID-19 Pandemic: Qualitative Analysis of Survey Responses. Int. J. Environ. Res. Public Health.

[10] Pascoe A., Paul E., Willis K., Smallwood N. (2022). Cross-sectional survey of COVID-19-related impacts on mental health of nurses: Occupational disruption, organisational preparedness, psychological harm, and moral distress. Contemp. Nurse.

[11] (2020). Survey of Nation’s Frontline Registered Nurses Shows Hospitals Unprepared For COVID-19 [press release]. National Nurses United.

[12] Office of the Assistant Secretary for Planning and Evaluation, U.S. Department of Health and Human Services (2022). Impact of the COVID-19 Pandemic on the Hospital and Outpatient Clinician Workforce: Challenges and Policy Responses.

[13] Thompson C.N., Baumgartner J., Pichardo C., Toro B., Li L., Arciuolo R., Chan P.Y., Chen J., Culp G., Davidson A. (2020). COVID-19 Outbreak—New York City, February 29–June 1, 2020. MMWR Morb. Mortal. Wkly. Rep..

[14] Glenza J.R.A., Villarreal A. (2020). “It’s what was happening in Italy”: The hospital at the center of New York’s COVID-19 Crisis. The Guardian.

[15] Silva D.I.J. (2020). “This is a war”: NYC doctors describe fight against coronavirus as cases surge. NBC News.

[16] Toner E.M.V., Hanfling D., Hick J., Daugherty Biddison L., Adalja A., Watson M., Evans L. (2020). Crisis Standards of Care: Lessons from New York CIty Hospitals' COVID-19 Experience. A Meeting Report.

[17] Keeley C., Jimenez J., Jackson H., Boudourakis L., Salway R.J., Cineas N., Villanueva Y., Bell D., Wallach A.B., Schwartz D.B. (2020). Staffing Up for The Surge: Expanding The New York City Public Hospital Workforce During The COVID-19 Pandemic. Health Aff..

[18] New York City Department of Health and Mental Hygiene (2022). Omicron Variant: NYC Report for January 13, 2022.

[19] Sellers F.S.C.A.E., Knowles H., Hawkins D. (2021). The delta variant is putting America's hospitals back in crisis mode. The Washington Post.

[20] New York City Department of Health and Mental Hygiene COVID-19 Data: Trends and Totals. Updated 25 September 2023. https://www.nyc.gov/site/doh/covid/covid-19-data-totals.page.

[21] Galanis P., Vraka I., Fragkou D., Bilali A., Kaitelidou D. (2021). Nurses’ burnout and associated risk factors during the COVID-19 pandemic: A systematic review and meta-analysis. J. Adv. Nurs..

[22] Kovner C., Raveis V.H., Van Devanter N., Yu G., Glassman K., Ridge L.J. (2021). The psychosocial impact on frontline nurses of caring forpatients with COVID-19 during the first wave of the pandemic in New York City. Nurs. Outlook.

[23] Vogus T.J., Sutcliffe K.M. (2007). The Safety Organizing Scale: Development and validation of a behavioral measure of safety culture in hospital nursing units. Med. Care.

[24] Gershon R.R., Karkashian C.D., Grosch J.W., Murphy L.R., Escamilla-Cejudo A., Flanagan P.A., Bernacki E., Kasting C., Martin L. (2000). Hospital safety climate and its relationship with safe work practices and workplace exposure incidents. Am. J. Infect. Control.

[25] SAS Institute Inc (2013). Base SAS^®^ 9.4 Utilities: Reference.

[26] Firew T., Sano E.D., Lee J.W., Flores S., Lang K., Salman K., Greene M.C., Chang B.P. (2020). Protecting the front line: A cross-sectional survey analysis of the occupational factors contributing to healthcare workers’ infection and psychological distress during the COVID-19 pandemic in the USA. BMJ Open.

[27] Ma Y., Faraz N.A., Ahmed F., Iqbal M.K., Saeed U., Mughal M.F., Raza A. (2021). Curbing nurses’ burnout during COVID-19: The roles of servant leadership and psychological safety. J. Nurs. Manag..

[28] Weick K.E., Kathleen M.S. (2015). Managing the Unexpected.

[29] Saleem M.S., Isha A.S.N., Mohd Yusop Y., Awan M.I., Naji G.M.A. (2021). Agility and Safety Performance among Nurses: The Mediating Role of Mindful Organizing. Nurs. Rep..

